# Comparison of the Substrate Preferences of ω3 Fatty Acid Desaturases for Long Chain Polyunsaturated Fatty Acids

**DOI:** 10.3390/ijms20123058

**Published:** 2019-06-22

**Authors:** Pushkar Shrestha, Xue-Rong Zhou, Sapna Vibhakaran Pillai, James Petrie, Robert de Feyter, Surinder Singh

**Affiliations:** CSIRO Agriculture & Food, Canberra, ACT 2601, Australia; Pushkar.Shrestha@csiro.au (P.S.); Sapna.Vibhakaranpillai@csiro.au (S.V.P.); James.Petrie@csiro.au (J.P.); Robert.Defeyter@csiro.au (R.d.F.); Surinder.Singh@csiro.au (S.S.)

**Keywords:** Omega-3 desaturase, long-chain polyunsaturated fatty acids, substrate specificity, EPA, DHA

## Abstract

Omega-3 long chain polyunsaturated fatty acids (ω3 LC-PUFAs) such as eicosapentaenoic acid (EPA; 20:5ω3) and docosahexaenoic acid (DHA; 22:6ω3) are important fatty acids for human health. These ω3 LC-PUFAs are produced from their ω3 precursors by a set of desaturases and elongases involved in the biosynthesis pathway and are also converted from ω6 LC-PUFA by omega-3 desaturases (ω3Ds). Here, we have investigated eight ω3-desaturases obtained from a cyanobacterium, plants, fungi and a lower animal species for their activities and compared their specificities for various C18, C20 and C22 ω6 PUFA substrates by transiently expressing them in *Nicotiana benthamiana* leaves. Our results showed hitherto unreported activity of many of the ω3Ds on ω6 LC-PUFA substrates leading to their conversion to ω3 LC-PUFAs. This discovery could be important in the engineering of EPA and DHA in heterologous hosts.

## 1. Introduction

Long chain polyunsaturated fatty acids such as arachidonic acid (ARA; 20:4ω6), eicosapentaenoic acid (EPA; 20:5ω3) and docosahexaenoic acid (DHA; 22:6ω3) are essential fatty acids for human health. Arachidonic acid is mainly located in the brain, skeletal muscles and liver, while EPA and DHA are rich in the brain, retina and skin. LC-PUFAs are divided into ω6 and ω3 LC-PUFAs, depending upon the positioning of the last double bond in the fatty acid chain, either at the sixth carbon from the terminal methyl end in ω6 fatty acids or at the third carbon in ω3 fatty acids. Beneficial effects on infant growth and development have been shown for ω6 LC-PUFAs such as ARA, but they have also been associated with blood coagulating (pro-thrombotic) and pain initiating (pro-inflammatory) properties, whereas ω3 LC-PUFAs have anti-thrombotic and anti-inflammatory properties. Thus, higher ω6/ω3 fatty acid ratios are related to several health problems such as obesity [1], diabetes, inflammatory–autoimmune diseases, mood disorders [2] and depression. On the other hand, the occurrence of low-level cardiovascular diseases in Inuit and Japanese populations has been attributed to the higher level of ω3/ω6 ratios in their bodies, which is believed to be due to the consumption of ω3 fatty acid-rich sea foods in their daily diet [3]. Recently, Simopoulus [1] has reported higher ω6/ω3 ratios in populations of the Western world. The ratio is alarmingly high in urban India due to the consumption mainly of land-sourced foods which are generally rich in ω6-PUFA and where there is little access to ω3 LC-PUFA rich marine foods. Clear health benefits have been shown for ω3 LC-PUFAs such as EPA and DHA for cardiovascular diseases, hyperlipidaemia, hypertension, inflammatory diseases, and their potential benefits for diabetes, mood disorders, cancer and Alzheimer disease have also been reported.

The human body produces only a low level of LC-PUFA from the consumption of shorter chained fatty acids from plant and animal sources. Because most land-based foods are richer in shorter chain C18 ω6 fatty acids than C18 ω3 fatty acids, their consumption leads to the production of a higher proportion of ω6 LC-PUFAs and a lower proportion of the healthier ω3 LC-PUFAs in the human body. Therefore, the Food and Agriculture Organization of the United Nations, the World Health Organization and the European Food Safety Authority have recommended an intake of a minimum of 250 mg of EPA + DHA daily to maintain good health [4]. Currently, marine fish and marine fish oil are the main sources of ω3 LC-PUFAs. However, fish do not synthesize LC-PUFA in their body but acquire these fatty acids by consuming ω3-PUFA-rich microalgae and phytoplankton. Certain marine flora have a set of special desaturases and elongases which convert short chain fatty acids to ω3 LC-PUFAs. In addition, some fatty acid desaturases have the ability to introduce a double bond at the ω3 position of different ω6 fatty acids and convert the ω6 PUFAs into ω3 PUFAs. These desaturases are therefore known as ω3-desaturases (ω3Ds).

The demand for ω3 LC-PUFAs is increasing for nutraceutical, pharmaceutical and aquaculture feeding purposes, while the marine fish stock, which is the main source, is declining globally. As an economical and sustainable alternative source of these fatty acids in oil seeds, fish oil-like levels of EPA [5] and DHA [6] were developed in the oil seed crop, camelina by the expression of a set of microalgal genes. There are some ω6 LC-PUFA intermediates in EPA camelina, while the levels of ω6 LC-PUFA are very low in DHA-camelina [6] and DHA-Arabidospsis [7] seeds, although they are rich in ω6 PUFA (18:2ω6) substrate. The role of the seed ω3Ds in converting the native and the engineered ω6 substrates into ω3 LC-PUFAs is not clear, and it is important to understand this mechanism. Nevertheless, ω3Ds have also shown important roles in the responses to different environmental stresses, such as temperature, drought, light, salinity, wounding and diseases [8].

Several ω3Ds have been identified in plants and microorganisms and they have shown diverse phylogeny, functional characteristics and substrate preferences. For example, ω3Ds obtained from *Fusarium* spp. [9] and *Mortierella alpina* [10] and expressed in yeast catalysed the desaturation of C18 ω6 PUFAs and, at lower activities, C20 ω6 PUFA substrates. In contrast, the ω3D from an EPA-rich fungus, *Saprolgenia diclina*, when expressed in yeast, did not recognize C18 substrates and was active only on C20 ω6 fatty acid substrates [11]. More broadly, the ω3D from another EPA-rich fungus, *Phytophthora infestans* [12], had greater activity on C20 and C22 fatty acid substrates than on C18 substrates but was active on all of those tested [13]. The FAT-1 ω3D of the EPA-containing animal species *Caenorhabditis elegans* [14] was highly active on C18 fatty acids as well as the C20 substrate, dihomo-gamma-linolenic acid (20:3ω6) [15]. Somewhat differently, when expressed in yeast, *Brassica napus* ω3D exhibited broad specificity for C16-C22 substrates [16] and the *Aspergillus nidulans* bifunctional oleoyl Δ12/linoleoyl ω3 desaturase converted C18 and C20 ω6 substrates to their respective ω3 fatty acids [17]. For others, the fungus *Neurospora crassa* produces ALA [18], possibly involving a ω3D, and *Synechocystis* sp. ω3D produces α-linolenic acid (18:3 ω3) and stearidonic acid (18:4 ω3) [19], but their ω3D specificities have not been investigated. Other special activities of ω3D had been also reported. For example, *M. alpina* 1S-4 ω3D also inserted a double bond at Δ15-position of medium chain fatty acid 16:2^9,12^, resulting in the formation of unusual 16:3^9,12,15^ [20]. Chang et al. [21] proposed that thraustochytrid ω3D could also work on odd-chain LC-PUFA 21:5 ω-5) leading to unusual 21:6 ω2.

However, our understanding of ω3D is still fragmentary [8], and the diversity of ω3D substrate specificity from various organisms is unclear or even unknown. A few of these ω3Ds have been tested in plant cells. Although the ability of ω3Ds to desaturate C18 and C20 substrates has been studied in a few species, their specificities for C22 PUFA substrates are still not understood.

Here, we have used a transient expression system in *N. benthamiana* leaves to investigate a range of eight ω3Ds obtained from a cyanobacterium, plants, fungi and the nematode *C*. *elegans* for their activities and compared their specificities for various C18, C20 and C22 LC-PUFA substrates. Our results showed an unexpected wide range of ω3D activity on various ω6 LC-PUFA substrates leading to their conversion to ω3 LC-PUFAs. Some of these activities were not reported previously. We also observed that these ω3Ds had different substrate preferences.

## 2. Results

### 2.1. Conversion of Linoleic Acid (LA, 18:2ω6) to α-Linolenic Acid (ALA, 18:3ω3) by ω3D

**Figure 1:**
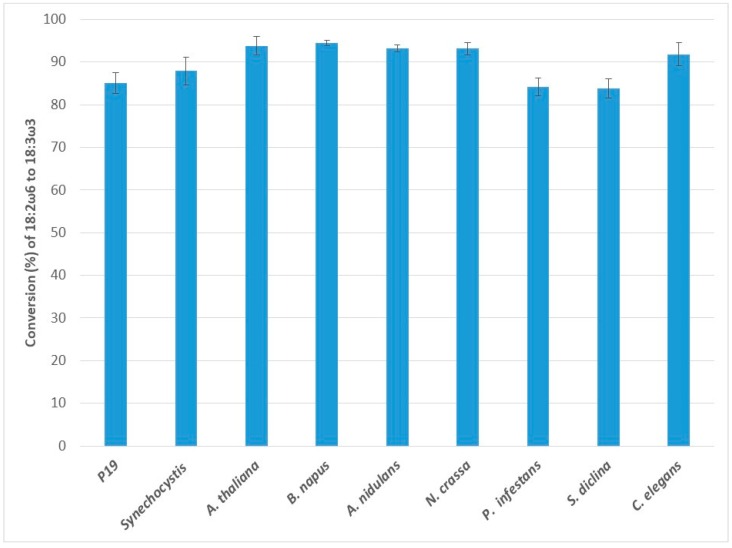
Enzymatic activities of transiently expressed ω3Ds from various sources in *N. benthamiana* leaves on 18:2ω6 substrate. ω3D activities were determined by measuring the conversion rate of endogenous 18:2ω6 substrate to 18:3ω3 in leaves expressing p19 silencing suppressor only as a control or the appropriate ω3D with p19. The error bars denote the standard deviations of the means from triplicate assays.

Eight individual ω3Ds were transiently expressed in *N. benthamiana* leaves in the presence of the p19 viral gene silencing suppressor to reduce co-suppression and extend ω3D production. The introduction of p19 alone was used as a control. *N. benthamiana* leaves possess endogenous ω3D activity which converts endogenously present linoleic acid to linolenic acid (Figure 1), and therefore all test samples were compared to the control. We first investigated the activities of transiently expressed ω3Ds by measuring the increase in the conversion rate of endogenous LA to ALA, compared to the p19-only infiltrated (control) tissues. The p19-only infiltrated tissues showed a 85.0 ± 2.5% conversion of LA to ALA, while the addition of *A. thaliana* ω3D (At-ω3D), *B. napus* ω3D (Bn-ω3D), *A. nidulans* ω3D (An-ω3D), *N*. *crassa* ω3D (Nc-ω3D), and *C*. *elegans* ω3D (Ce-ω3D) exhibited 93.8 ± 2.2%, 94.5 ± 0.6%, 93.2 ± 0.8%, 93.1 ± 1.4%, and 91.8 ± 2.8% conversions of LA, respectively, indicating the expression of the introduced ω3D genes in the leaves and their activities for LA (Figure 1). Among all these, Bn-ω3D had the highest conversion rate for LA, which was 9.5% higher than the endogenous ω3D activity of the leaves (p19 only). However, the conversion rates with *P. infestans* ω3D (Pi-ω3D; 84.1 ± 2.0%) and *S. diclina* ω3D (Sd-ω3D; 83.8 ± 2.2%) were essentially the same as the control, indicating little or no activity for those enzymes on LA. *Synechocystis* ω3D (Ss-ω3D; 87.9 ± 3.3%) showed a slightly increased conversion of LA.

### 2.2. Conversion of Gamma-Linolenic Acid (18:3ω6) to Stearidonic Acid (18:4ω3)

**Figure 2:**
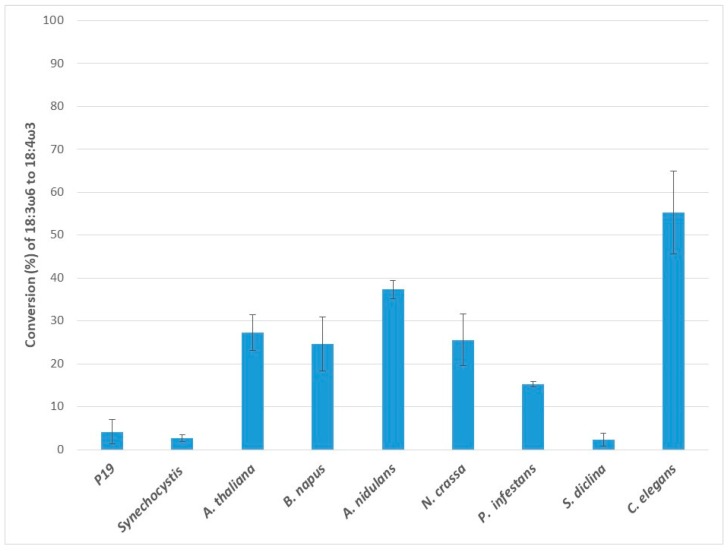
Enzymatic activities of transiently expressed ω3Ds from various sources in *N. benthamiana* leaves on 18:3ω6 substrate. ω3D activities were determined by measuring the conversion rate of provided 18:3ω6 substrate to 18:4ω3 in leaves expressing p19 silencing suppressor only as a control or the appropriate ω3D with p19. The error bars denote the standard deviations of the means from triplicate assays.

Application of gamma-linolenic acid (GLA) to the ω3D expressing leaf tissues resulted in the production of stearidonic acid, indicating the ω3D activities on another C18 ω6 substrate, GLA (Figure 2). The conversion rate of the endogenous *N. benthamiana* leaf ω3D was 4.2 ± 2.9%. The transient expression of exogenous ω3Ds clearly showed higher conversion rates for GLA than the endogenous ω3D in the control and were 55.2 ± 9.7%, 37.3 ± 2.2%, 25.5 ± 6.0%, 15.3 ± 0.6%, 27.2 ± 4.2% and 24.6 ± 6.3% for Ce-ω3D, An-ω3D, Nc-ω3D, Pi-ω3D, At-ω3D and Bn-ω3D, respectively. The conversion rate of Ce-ω3D for GLA was the highest, being 13.1-fold higher than the endogenous conversion efficiency of the leaf ω3D. Sd-ω3D and Ss-ω3D showed no activity on GLA; i.e., no increase was observed in the conversion rate compared to the p19 control.

### 2.3. Conversion of Dihomo Gamma-Linolenic Acid (20:3ω6) to Eicosatetraenoic Acid (20:4ω3)

**Figure 3:**
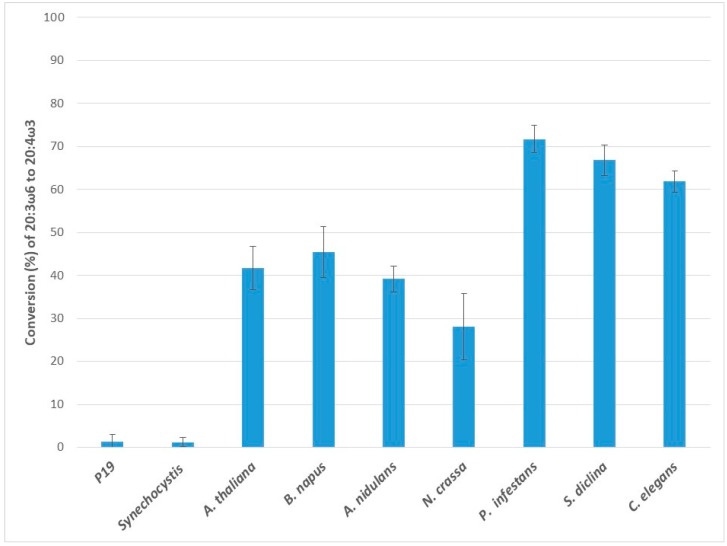
Enzymatic activities of transiently expressed ω3Ds from various sources in *N. benthamiana* leaves on 20:3ω6 substrate. ω3D activities were determined by measuring the conversion rate of provided 20:3ω6 substrate to 20:4ω3 in leaves expressing p19 silencing suppressor only as a control or the appropriate ω3D with p19. The error bars denote the standard deviations of the means from triplicate assays.

The application of dihomo gamma-linolenic acid (DGLA) to the ω3D-expressing leaf tissues resulted in the production of eicosatetraenoic acid (ETA), indicating activities of ω3D on the C20 ω6 substrate (Figure 3). The endogenous ω3D had a low conversion rate for DGLA. Except for Ss-ω3D, the other seven ω3Ds showed a high level of activity for DGLA compared to the endogenous activity of the leaves (1.4 ± 1.7%). Pi-ω3D showed a very high conversion rate for DGLA to ETA (71.7 ± 3.2%), in contrast to its lower level of activity with GLA. Although GLA activity was absent with Sd-ω3D, it showed a strong preference for DGLA (66.8 ± 3.6%), and Ce-ω3D (61.8 ± 2.5%), Nc-ω3D (28.1 ± 7.7%) and An-ω3D (39.2 ± 3.0%) also showed high DGLA activities. Interestingly, the plant ω3Ds, Bn-ω3D and At-ω3D revealed conversion efficiencies of 45.5 ± 6.0 and 41.7 ± 5.0%, respectively, for the C20 substrate DGLA, which is not a native substrate in plants. Among the enzymes, Pi-ω3D showed the highest conversion rate for DGLA. Again, just like its GLA response, Ss-ω3D did not show any activity with DGLA.

### 2.4. Conversion of Arachidonic Acid (20:4ω6) to Eicosapentaenoic Acid (20:5ω3)

**Figure 4:**
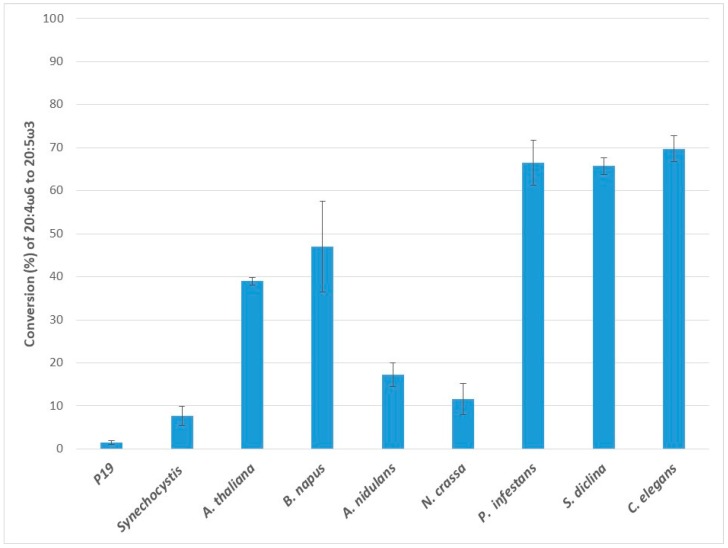
Enzymatic activities of transiently expressed ω3Ds from various sources in *N. benthamiana* leaves on 20:4ω6 substrate. ω3D activities were determined by measuring the conversion rate of provided 20:4ω6 substrate to 20:5ω3 in leaves expressing p19 silencing suppressor only as a control or the appropriate ω3D with p19. The error bars denote the standard deviations of the means from triplicate assays.

When arachidonic acid (ARA) was supplied, all eight ω3Ds investigated were able to convert it to EPA (Figure 4). Again, the endogenous ω3D in p19 control had a low conversion rate for ARA. The highest activity level was observed for Ce-ω3D with the conversion rate of 69.7 ± 3.0%, followed by Pi-ω3D (66.4 ± 5.2%) and Sd-ω3D (65.7 ± 2.0%), while the plant ω3Ds, Bn-ω3D and At-ω3D exhibited 47.0 ± 10.5% and 39.0 ± 0.9% conversion of ARA. Nc-ω3D (11.6 ± 3.7%) and An-ω3D (17.2 ± 2.7%) had lower levels of conversion with ARA. Ss-ω3D had a 7.6 ± 2.2% conversion rate for ARA, in contrast to an absence of activities with GLA and DGLA. 

### 2.5. Conversion of Docosatetraenoic Acid (22:4ω6) to Docosapentaenoic Acid (22:5ω3)

**Figure 5:**
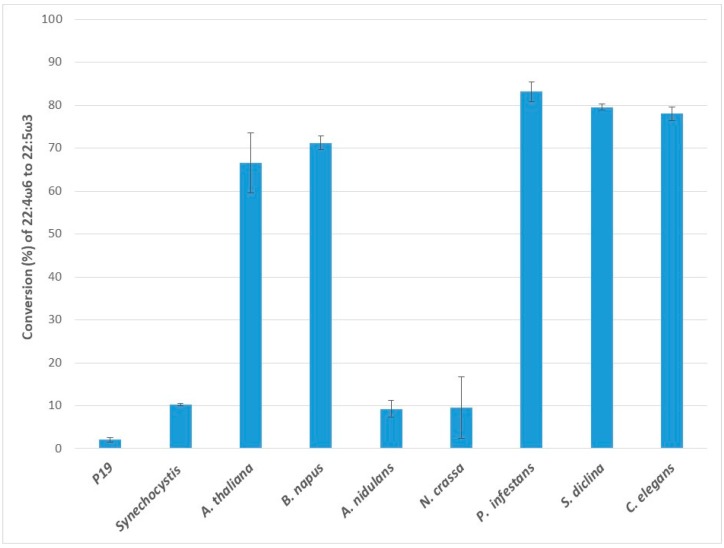
Enzymatic activities of transiently expressed ω3Ds from various sources in *N. benthamiana* leaves on 22:4ω6 substrate. ω3D activities were determined by measuring the conversion rate of provided 22:4ω6 substrate to 22:5ω3 in leaves expressing p19 silencing suppressor only as a control or the appropriate ω3D with p19. The error bars denote the standard deviations of the means from triplicate assays.

When the C22:4ω6 fatty acid docosatetraenoic acid (DTA) was supplied, all the ω3Ds tested were observed to desaturate DTA to the ω3 product docosapentaenoic acid (DPA) (Figure 5). The endogenous ω3D in p19 control had little activity on the provided C22 substrate. High levels of conversion were observed for Pi-ω3D (83.2 ± 2.3%), Sd-ω3D (79.5 ± 0.7%), and Ce-ω3D (78.0 ± 1.5%), as well as the plant ω3Ds, Bn-ω3D (71.2 ± 1.6%) and At-ω3D (66.6 ± 7.0%). Ss-ω3D (10.2 ± 0.3%), Nc-ω3D (9.6 ± 7.2%) and An-ω3D (9.3 ± 2.0%) showed lower rates of DTA conversion. 

### 2.6. Conversion of Docosapentaenoic Acid-6 (22:5ω6) to Docosahexaenoic Acid (22:6ω3)

**Figure 6:**
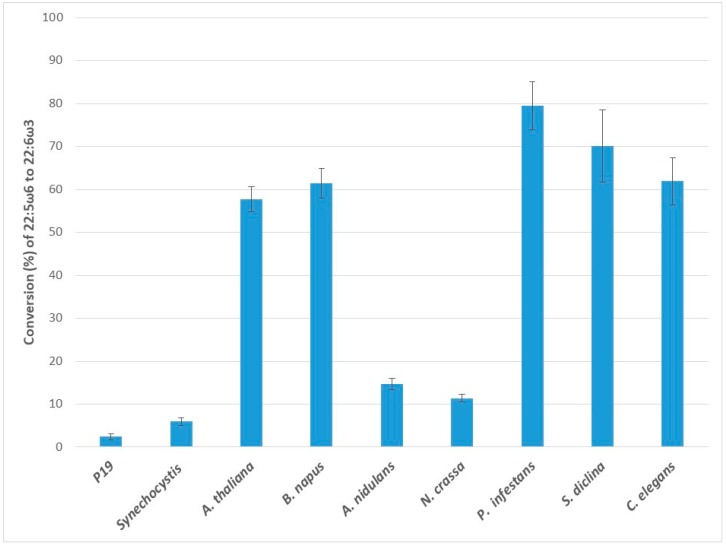
Enzymatic activities of transiently expressed ω3Ds from various sources in *N. benthamiana* leaves on 22:5ω6 substrate. ω3D activities were determined by measuring the conversion rate of provided 22:5ω6 substrate to 22:6ω3 in leaves expressing p19 silencing suppressor only as a control or the appropriate ω3D with p19. The error bars denote the standard deviations of the means from triplicate assays.

When the C22:5ω6 fatty acid docosapentaenoic acid (DPAω6) was supplied, all the ω3Ds studied here converted it to DHA (Figure 6). The endogenous ω3D in p19 control had minor activity on the provided C22 substrate, as observed above. The highest rate of conversion was seen for Pi-ω3D (79.5 ± 5.6%), followed by Sd-ω3D (70.1 ± 8.4%), Ce-ω3D (61.9 ± 5.5%), Bn-ω3D (61.4 ± 3.5%) and At-ω3D (57.7 ± 2.9%). An-ω3D (14.7 ± 1.4%), Nc-ω3D (11.4 ± 0.8%) and Ss-ω3D (5.9 ± 0.9%) had lower conversion rates for DPAω6. 

### 2.7. Competition Among ω6 Fatty Acid Substrates for ω3Ds

**Figure 7:**
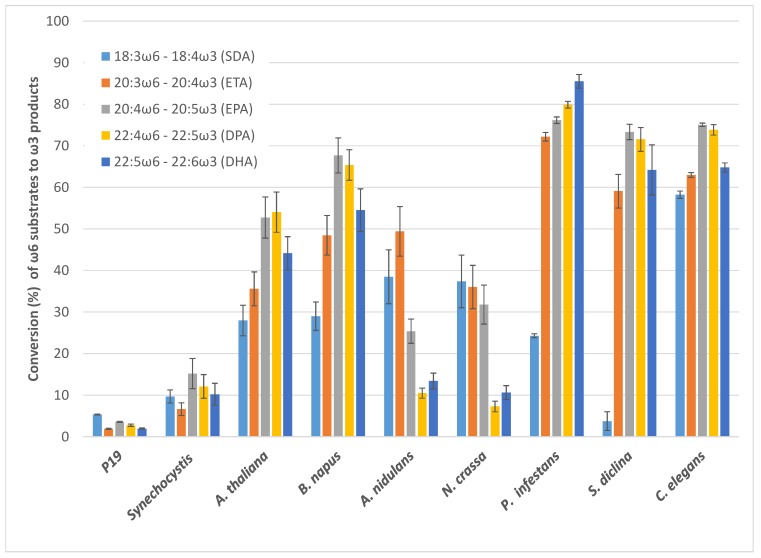
Competition for ω6-fatty acid substrates (18:3ω6, 20:3ω6, 20:4ω6, 22:4ω6, 22:5ω6) by ω3-desaturases from various sources transiently expressed in *N. benthamiana*. Error bars represent the standard deviations of the means from triplicate assays.

In nature, multiple fatty acid substrates are available to a ω3D at the same time. The activity for one substrate might be affected by the presence of other substrates, and the enzyme might have preferential selectivity for one substrate over others. Here, we attempted to measure the conversion efficiency of each substrate during competition among a pool of five different substrates (18:3ω6, 20:3ω6, 20:4ω6, 22:4ω6, 22:5ω6). Unlike the individual substrate assays, Ss-ω3D showed the highest conversion rate of 20:4ω6 up to 15.2 ± 3.6% among the pool of substrates followed by 22:4ω6, 22:5ω6, 18:3ω6 and 20:3ω6 (Figure 7). The conversion rates of both At-ω3D and Bn-ω3D for 20:4ω6 (52.7 ± 5.0%, 67.7 ± 4.2%) and 22:4ω6 (54.0 ± 4.8%, 65.4 ± 3.7%) during substrate competition were the highest among all the substrates, followed by 22:5ω6, 20:3ω6 and 18:3ω6). An-ω3D and Nc-ω3D demonstrated the preference for the C18 or C20 substrates. The An-ω3D activities for 20:3ω6 was the highest among these five substrates with the conversion rate of 49.4 ± 6.0%, followed by the activities for 18:3ω6 and 20:4ω6. Nc-ω3D had a similar higher preference for 18:3ω6, 20:3ω6 and 20:4ω6, all above a 30% conversion rate. Both An-ω3D and Nc-ω3D had conversion rates for 22:4ω6 and 22:5ω6 of only around 10%. Pi-ω3D had higher activities for both C20 and C22 substrates with a conversion rate above 70%, but low activity for 18:3ω6 with a conversion rate of 24.3 ± 0.5%. Sd-ω3D also showed a higher preference for both C20 and C22 substrates, with a conversion rate at 60% or above, while a very low preference for 18:3ω6, with a conversion rate of 3.8 ± 2.3%. Interestingly, Ce-ω3D showed strong activities for all the tested C18, C20 and C22 substrates, ranging from 58% to 75%. Among these eight ω3Ds, Ce-ω3D had the highest conversion rate for 18:3ω6.

## 3. Discussion 

To compare the ω3D enzymes on different ω6 fatty acid substrates, we used a *N. benthamiana* transient expression system [22] and co-infiltrated genetic constructs encoding the enzymes with the fatty acids prepared as ammonium salts. Prior to GC analysis, leaf fatty acid methyl esters (FAMEs) were purified by thin layer chromatography (TLC) to remove non-fatty acid contaminants that otherwise co-eluted with some of the FAMEs. To the best of our knowledge, this is the first report of an efficient assay of ω3D activity in leaves. The leaf system has advantages over yeast expression systems for ω3Ds which often fail to show activity or only reveal low activity levels in yeast cells [16,23]. Applying this approach, we were able to observe high and consistent levels of desaturase activity in the *N. benthamiana* leaf-based system and examine the substrate preferences of the different ω3Ds. In general, the ω3D conversion efficiencies observed here were higher than those observed in other studies in yeast cells, and we clearly demonstrated the diversity in ω3D activity and their broad specificity for various fatty acid substrates. 

It was notable that among the eight ω3Ds studied here, the two plant ω3Ds, At-ω3D and Bn-ω3D, revealed the highest activities for the endogenous 18:2ω6 substrate. The LC-PUFA biosynthesis pathway can use both 18:2ω6 and 18:3ω3 as precursors to produce ω6 and ω3 LC-PUFAs. Conversion of 18:2ω6 to 18:3ω3 would increase the ω3/ω6 ratio. The higher activity of At-ω3D and Bn-ω3D for 18:2ω6 might imply that engineering the LC-PUFA biosynthesis pathway in these plants has the potential to enhance ω3 LC-PUFA production. In contrast, Pi-ω3D and Sd-ω3D from EPA-rich species did not exhibit activity on 18:2ω6. Similar observations were made by others in yeast cells [11]. 

The specificity of the eight ω3Ds for the ω6 fatty acid substrates tested is summarised in Supplementary Figure S1. For the 18:3ω6 substrate (GLA), Ce-ω3D had the highest activity among all the ω3Ds investigated, while the plant ω3Ds, At-ω3D and Bn-ω3D, had about half of the Ce-ω3D activity. The desaturases Pi-ω3D and Sd-ω3D obtained from EPA-rich fungi and the animal desaturase Ce-ω3D revealed high activity levels for the C20 substrates, 20:3ω6 (DGLA) and 20:4ω6 (ARA), and higher still for the C22 substrates, 22:4ω6 (DTA) and 22:5ω6 (DPAω6). Recently, Yilmaz et al. [13] also demonstrated the activities of Pi-ω3D for C18, C20 and C22 fatty acid substrates in yeast, but in contrast to our study in leaf, observed lower activities to C22 than C20 fatty acids. On the other hand, Sd-ω3D, when expressed in yeast, exclusively desaturated C20 fatty acid substrates (5% conversion of 20:3ω6 to 20:4ω3 and 26% conversion of 20:4ω6 to 20:5ω3) [11]. However, there was no conversion of 18:2ω6 or 18:3ω6 to ω3 products. We also observed that the animal Ce-ω3D had greater activities for C20 and C22 substrates than for 18:3ω6, and that the level of activity was as high as for Pi-ω3D and Sd-ω3D. This contrasted with the observations of [15] in a yeast expression system, where Ce-ω3d had lower activity for 20:4ω6 than 20:3ω6 and 18:3ω6. 

Although higher plants are devoid of C20 and C22 LC-PUFA substrates, it is interesting that At-ω3D and Bn-ω3D showed high activity levels with all C20 and C22 substrates tested. Also, Reed [16] demonstrated the broad specificity of Bn-ω3D for C18-C22 fatty acids when expressed in yeast, although the activities were at low levels. In contrast, the heterologous expression in yeast of another plant ω3D from *Linum usitatissimum* did not result in ω3D activity with either 18:3ω6 or 20:4ω6 [23]. Here, the plant ω3D activities observed were higher for C22 substrates than for C20 and C18 substrates and were comparable to the EPA-rich fungal ω3Ds or the animal ω3D. The activity of plant ω3Ds was also evident from the conversion of endogenous 18:2ω6 to 18:3ω3 and 20:2ω6 to 20:3ω3. These plant endogenous ω3Ds might have roles in the conversion of ω6 PUFA to ω3 PUFA in DHA-producing engineered seeds, resulting in reduced levels of ω6 PUFA [6,7]. 

In contrast, two of the fungal desaturases, An-ω3D and Nc-ω3D, which share close phylogeny with each other, have activities for C18 substrates that are similar to or even higher than the other ω3Ds, whereas the activities were lower for C20 substrates and much lower for C22 substrates, demonstrating a clear deviation in substrate specificity for the fungal ω3Ds. The cyanobacterial Ss-ω3D was unable to desaturate 18:3ω6 and 20:3ω6 substrates, but it demonstrated some activities for 20:4ω6, 22:4ω6 and 22:5ω6. 

The substrate competition assay where multiple ω6 fatty acids were supplied to these ω3Ds showed similar patterns to the individual substrate assays, although 20:4ω6 and 22:4ω6 were the preferred substrates in the mixture for a majority of the tested enzymes, while 22:4ω6 was preferred in the single substrate assays. When engineering the LC-PUFA biosynthesis pathway into crops, there would be substrate competition from different ω6 intermediates; thus, the preference of ω3Ds for these substrates would reflect the in vivo activities better than single substrate assays. One interesting observation was that the plant ω3Ds, At-ω3D and Bn-ω3D, had similar activity levels, and their specificities were comparable to the EPA-rich fungal Pi- and Sd-ω3Ds and the animal Ce-ω3D, all of which exhibited higher activity for C20 and C22 LC-PUFA substrates than for C18 substrates. These have the potential to increase ω3/ω6 ratios when seeking to engineer LC-PUFAs into plants for health benefits.

## 4. Materials and Methods

### 4.1. Materials and Chemicals

Fatty acid substrates were purchased from NuCheck Inc. (Elysian, MN, USA) and ammonium salts of the fatty acids were synthesised in our laboratory. Briefly, 5 mg of fatty acid was dispersed in 0.5 mL of 2 M ammonia in a 2 mL glass vial using a probe sonicator (Branson, Switzerland) three times for 3 s each, vortexing between sonications. The mixture was then incubated at 60 °C for 20 min. The ammonia solution was evaporated under a flow of nitrogen on a 60 °C hot plate, and 1 mL of potassium–phosphate buffer was added to the vial. The salt was solubilised in the buffer by sonication. The concentration of fatty acid salt was estimated by injecting its fatty acid methyl ester into a gas chromatograph. For the preparation of the fatty acid methyl ester (FAME) of the fatty acid salt, 5 µL of the salt solution and 5 µg of heptadecanoic acid (C17:0 as internal standard) in toluene were mixed in 100 µL of methanol in a 2 mL glass vial, and the methanol/water mixture was evaporated under a gentle flow of nitrogen, placing the vial on a 40 °C hot plate. The fatty acid salt was incubated in 0.3 mL 1N methanolic-HCl (Supelco, Castle Hill, New South Wales, Australia) at 80 °C for 2 h. After cooling the solution to room temperature, 0.3 mL of 0.9% NaCl and 0.3 mL of hexane were added and then mixed for 5 min. The mixture was centrifuged for 5 min at 1700 × g, the upper phase of the FAME was transferred to a glass insert and the solvent was evaporated under a flow of nitrogen. The FAME was resuspended in 30 µL of hexane and analysed by GC as described [24].

### 4.2. Gene Constructs 

The ω3D sequences were obtained from *Synechocystis* sp. [25], PMB 26:249-263), *A. thaliana* (Accession# P48623, [26]), *B. napus* (Accession# L01418, [27]), *A. nidulans* (Sequence 5 from US Patent Application No. 20060156435), *N. crassa* (Sequence 3 from US Patent Application No. 20060156435), *P. infestans* (SEQ ID NO:2 from US Patent No. 7,777,098), *S. diclina* (SEQ ID NO:26 from US Patent No. 7,211,656) and *C. elegans* (Accession# L41807; [28]), and are designated as Ss-ω3D, At-ω3D, Bn-ω3D, An-ω3D, Nc-ω3D, Pi-ω3D, Sd-ω3D and Ce-ω3D, respectively. The coding sequences were synthesised at GeneArt (Thermo Fisher Scientific) with codon optimisation and cloned into the binary vector pJP3343 [29] under control of the CaMV 35S promoter. 

### 4.3. Transient Expression in N. benthamiana Leaf and Fatty Acid Analysis

*Agrobacterium tumefaciens* strain AGL1 was separately transformed with the gene constructs. Transformed cells were co-infiltrated with AGL1 containing a viral silencing protein (p19) gene into *N. benthamiana* leaves as described before [22] with some modifications. In brief, AGL1 cultures were grown overnight at 28 °C in LB broth containing appropriate antibiotics. Acetosyringone (0.1 mM) was added to the cultures, which were grown for a further 3 h before the cells were pelleted down and resuspended in infiltration buffer (5 mM 4-morpholineethanesulfonic acid, 5 mM MgSO_4_). 

The solubilized fatty acid salts were added in the infiltration buffer at the final concentration of 1 mM each for single substrate assay or 0.5 mM each for competition assay with each Agrobacterium culture at OD_600_ of 0.1. Approximately 0.1 mL of the appropriate mixture was infiltrated in each spot with a needleless syringe on the undersides of leaves of five-week-old plants. After 3 days in a growth chamber, the infiltrated leaf tissues were collected into 2 mL glass vials and washed once with 0.2% tween and twice with water to wash off any remaining fatty acid substrate(s) from the leaf surface. The leaf samples were dried in a freeze dryer overnight, and fatty acid methyl esters were prepared as mentioned above. To remove non-fatty acid leaf contaminants from the FAMEs, FAME samples were applied to a TLC plate (Silica gel-60, MERCK, Castle Hill, New South Wales, Australia) and run in a mixture of hexane/diethyl ether/acetic acid (80/20/1, *v*/*v*/*v*). FAME bands were viewed under UV after spraying the plates with 0.01% primuline in acetone/water (8/2, *v*/*v*). The bands were collected into GC vials and FAMEs were extracted from the silica using hexane/ diethyl ether (1/3, *v*/*v*) and analysed by GC as described [24].

## 5. Conclusions

In conclusion, ω3Ds show activity on a range of C18-C22 ω6 fatty acids and a diversity of substrate preference and level of activity in producing ω3 LC-PUFAs. This will be important for EPA and DHA production in heterologous hosts. Of particular interest, the endogenous plant ω3Ds had previously unsuspected activities on a wide range of ω6 fatty acid substrates, especially on the LC-PUFA substrates. This would enhance the ability to engineer oil crops as alternative sources of ω3 LC-PUFAs.

## Supplementary Materials

Supplementary materials can be found at https://www.mdpi.com/1422-0067/20/12/3058/s1.

## Abbreviations

ALA	α-linolenic acid (18:3ω3)
An	*Aspergillus nidulans*
ARA	arachidonic acid (20:4ω6)
At	*Arabidopsis thaliana*
Bn	*Brassica napus*
Ce	*Caenorhabditis elegans*
DGLA	dihomo gamma-linolenic acid (20:3ω6)
DHA	docosahexaenoic acid (22:6ω3)
DPA	docosapentaenoic acid (22:5ω3)
DPA-6	docosapentaenoic acid (22:5ω6)
DTA	docosatetraenoic acid (22:4ω6)
EPA	eicosapentaenoic acid (20:5ω3)
ETA	eicosatetraenoic acid (20:4ω3)
FAME	fatty acid methyl ester
GC	gas chromatography
GLA	gamma-linolenic acid (18:3ω6)
LA	linoleic acid (18:2ω6)
LC-PUFA	long chain-polyunsaturated fatty acid
Nc	*Neurospora crassa*
ω3D	omega-3 desaturase
Pi	*Phytophthora infestans*
SDA	stearidonic acid
Ss	*Synechocystis* sp.
Sd	*Saprolegnia diclina*
TLC	thin layer chromatography
