# Supplementary material for: Comparison of the Substrate Preferences of ω3 Fatty Acid Desaturases for Long Chain Polyunsaturated Fatty Acids
Source: Int J Mol Sci. 2019 Jun 22;20(12):3058. doi: 10.3390/ijms20123058 (PMC6627408; doi:10.3390/ijms20123058)
Supplement: Supplementary file 1 [file ijms-20-03058-s001.pdf]

International Journal of
Molecular Sciences

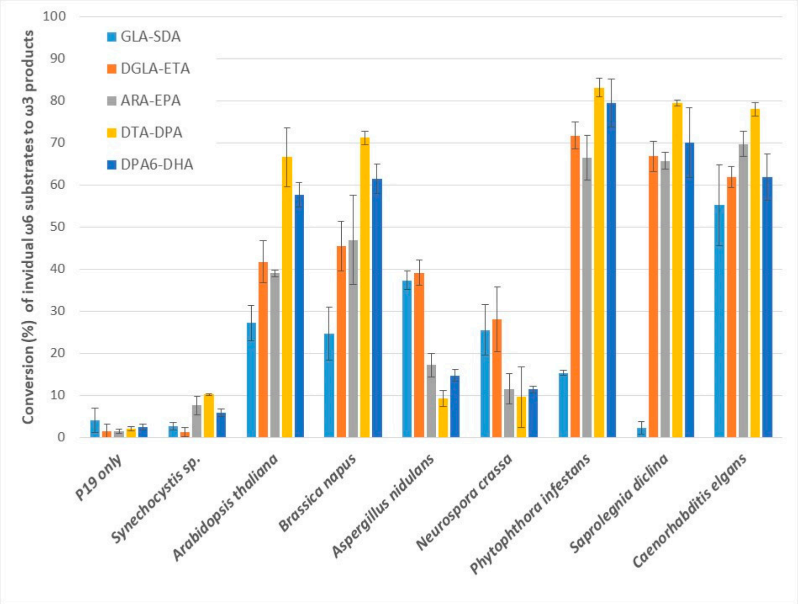

Supplementary Figure S1. Summary of ω3D enzymatic activities with the supplied ω6 fatty acid salt substrates. ω3D activities are shown as conversion rates determined by the amounts of ω3 fatty acid products produced from the total amount of ω6 fatty acid substrates. Activity from the p19-only control represents the leaf endogenous ω3D activity. The error bars denote standard deviations of the means from triplicate assays.
